# Imaging response to immune checkpoint inhibitors in patients with advanced melanoma: a retrospective observational cohort study

**DOI:** 10.3389/fonc.2024.1385425

**Published:** 2024-05-31

**Authors:** Mehul Gupta, Igor Stukalin, Daniel E. Meyers, Daniel Y. C. Heng, Jose Monzon, Tina Cheng, Vishal Navani

**Affiliations:** ^1^ Cumming School of Medicine, University of Calgary, Calgary, AB, Canada; ^2^ Tom Baker Cancer Centre, Calgary, AB, Canada

**Keywords:** melanoma, immunotherapy, Response Evaluation Criteria in Solid Tumors (RECIST), survival, prognosis

## Abstract

**Background:**

The association between objective imaging response and first line immune checkpoint inhibitor (ICI) therapy regimes in advanced melanoma remains uncharacterized in routine practice.

**Methods:**

We conducted a multi-center retrospective cohort analysis of advanced melanoma patients receiving first line ICI therapy from August 2013-May 2020 in Alberta, Canada. The primary outcome was likelihood of RECIST v1.1 assessed objective imaging response between patients receiving anti-programmed cell death protein 1 (anti-PD1) monotherapy and those receiving combination ipilimumab-nivolumab. Secondary outcomes were identification of baseline characteristics associated with non-response and the association of imaging response with overall survival (OS) and time to next treatment (TTNT).

**Results:**

198 patients were included, 41/198 (20.7%) had complete response, 86/198 (43.4%) had partial response, 23/198 (11.6%) had stable disease, and 48/198 (24.2%) had progressive disease. Median OS was not reached (NR) (95% CI 49.0-NR) months for complete responders, NR (95%CI 52.9-NR) months for partial responders, 33.7 (95%CI 15.8-NR) months for stable disease, and 6.4 (95%CI 5.2–10.1) months for progressive disease (log-rank p<0.001). Likelihood of objective imaging response remained similar between anti-PD1 monotherapy and ipilimumab-nivolumab groups (OR 1.95 95%CI 0.85–4.63, p=0.121). Elevated LDH level (OR 0.46; 95%CI 0.21–0.98, p=0.043), mucosal primary site (OR 0.14; 95%CI 0.03–0.48, p=0.003), and BRAF V600E mutation status (OR 0.31; 95%CI 0.13–0.72, p=0.007) were associated with decreased likelihood of response.

**Conclusion:**

No significant difference in likelihood of imaging response between anti-PD1 monotherapy and combination ipilimumab-nivolumab was observed. Elevated LDH level, mucosal primary site, and BRAF V600E mutation status were associated with decreased likelihood of response. Given that pivotal clinical trials of ipilimumab-nivolumab did not formally compare ipilimumab-nivolumab with nivolumab monotherapy, this work adds context to differences in outcomes when these agents are used. These results may inform treatment selection, and aid in counseling of patients treated with first-line ICI therapy in routine clinical practice settings.

## Introduction

Immune checkpoint inhibitor (ICI) therapy has dramatically improved survival outcomes among patients with advanced melanoma (1, 2). Currently established treatment regimens consist of anti-programmed cell death protein 1 (anti-PD1) monotherapies such as pembrolizumab or nivolumab, or combination nivolumab with ipilimumab, a cytotoxic T-cell lymphocyte-antigen 4 inhibitor (anti-CTLA4) (3). Despite improvements in survival outcomes using these regimes, the majority patients treated with first-line ICI therapy eventually develop resistance, resulting in suboptimal treatment outcomes (4).

Though there remain significant challenges with using the Response Evaluation Criteria in Solid Tumors (RECIST) version 1.1 to assess for response in the setting of immunotherapy (5–9), these guidelines played a central role in pivotal studies of ICI therapy in advanced melanoma and are still utilized to define surrogate end points across observational studies and clinical trials. Though a positive association between objective imaging response and durable survival among advanced melanoma patients receiving first-line ICI therapy has been well established in clinical trial populations (10–12), there remains a paucity of studies examining this association in a routine clinical practice setting.

Evidence in advanced melanoma patients receiving first-line ICI therapy suggests that survival varies markedly between patients with only 40% of those treated with anti-PD1 therapy being alive at three years (13). Additionally, clinical trial data suggests that only 30–40% of patients treated with anti-PD1 monotherapy (14–17), and 50–60% of patients treated with combination ipilimumab-nivolumab therapy (4, 18) experience objective imaging response (complete response or partial response as per RECIST v1.1 criteria). Given this heterogenous response to conventional first-line ICI therapy in advanced melanoma patients, accessible biomarkers to predict objective imaging response would be helpful to tailor treatment selection in this context. This is especially true if reducing tumour burden is necessary to prolong life and palliate related symptoms. Many different markers, including those focusing on tumour characteristics and other clinical parameters, have been proposed as putative candidate factors (19–23). However, attempts to discriminate patient populations using these markers are infrequent, with a lack of studies focused on patients with advanced melanoma treated with first-line ICI therapies in a real-world setting (24–27). Given this evidence gap, we utilize a real-world observational cohort to compare the likelihood of objective imaging response between first-line ICI therapy regimes and identify accessible baseline characteristics associated with objective imaging response in advanced melanoma.

## Materials and methods

### Study design and patient population

The Alberta Immunotherapy Database (AID) is a multi-center, observational cohort study that retrospectively captures baseline demographic, histological, clinical, laboratory, and imaging data utilizing a standardized data collection template of patients receiving immunotherapy in 2 academic and 5 community centers in Alberta, Canada (28). This sub-study examined patients with advanced melanoma receiving treatment between August 2013 and May 2020. Statistical analyses were performed in September 2023. Centralized institutional review board ethics approval from the Health Research Ethics Board of Alberta, Canada was obtained prior to undertaking data collection. This study followed the Strengthening the Reporting of Observational Studies in Epidemiology (STROBE) reporting guideline for cohort studies.

Inclusion criteria for this study included histologically confirmed locally advanced or metastatic melanoma (stage III unresectable or IV metastatic). All patients must have received first-line ICI therapy with either anti-PD1 agent monotherapy (nivolumab or pembrolizumab) or combination ipilimumab-nivolumab. Additionally, all patients included in the study were required to have an evaluable imaging response, ascertained by the presence of baseline CT imaging followed by one or more sets of repeat CT imaging while on ICI therapy. Patients not meeting the above inclusion criteria were excluded from the primary analysis. Additionally, patients with a uveal primary site were excluded from this analysis given historically poor response to immune checkpoint inhibitors, their exclusion from the pivotal CheckMate 067 study (11) as well as the lack of an approved indication for the latter for patients with metastatic uveal melanoma in Alberta.

### Outcome measures

The primary outcome of interest was the difference in investigator assessed imaging response per RECIST v1.1 guidelines (29) between type of first-line ICI therapy received (anti-PD1 monotherapy vs combination ipilimumab-nivolumab). Secondary outcomes of interest included the relationship between objective imaging response and key time to event endpoints such as overall survival (OS) and time to next treatment (TTNT), as well as the relationship between baseline characteristics and likelihood of objective imaging response.

Responders were classified as patients that experienced an investigator assessed best overall response (BOR) as per the RECIST v1.1 guidelines (29) of complete response (CR) or partial response (PR) while non-responders were those who experienced a BOR of stable disease (SD) or progressive disease (PD). For all analyses, OS was defined as the time from first-line ICI therapy initiation to death or censored at last follow up. TTNT was defined as the time from first-line ICI therapy initiation to subsequent systemic anti-cancer therapy initiation, death, or censored at last follow up. Baseline clinical and tumour characteristics were collected utilizing a standardized data collection template as described previously (28). Characteristics with a putative association with imaging response in the literature were selected *a priori* and dichotomized utilizing standard cut-offs for further analyses.

### Statistical analysis

Baseline demographic, clinical, laboratory, and treatment characteristics were stratified by imaging response and described with frequencies and proportions for categorical variables and median with IQR for numeric variables. Statistical analysis for differences in characteristics between subgroups was conducted using two-sided Fisher exact tests for discrete variables and non-parametric Mann-Whitney tests for continuous variables. Multivariable adjusted logistic regression analysis was used to determine potential associations between multiple baseline characteristics of interest as well as type of first-line ICI therapy and objective imaging response. Time-to-event end points, such as OS and TTNT, were evaluated using the Kaplan-Meier method, with Cox proportional hazards (CoxPH) regression for statistical analysis. The case-deletion method was used when missing data was encountered. All statistical tests were 2-sided with a significance threshold of *P* ≤ 0.05. All statistical analyses were carried out in R v 4.0.2 (R foundation for statistical computing, Vienna, Austria).

## Results

### Baseline characteristics

Of the 497 patients included in the AID metastatic melanoma database, a total of 316 were treated with first-line anti-PD1 monotherapy or ipilimumab-nivolumab. Of these, a total of 198 patients (62.7%) had evaluable imaging response data as well as baseline clinicopathologic variables of importance and were retained for analyses in this study (**Supplementary Figure 1**). Medial follow-up time from ICI therapy initiation was 36.10 months (IQR 26.10–43.77 months) using the reverse Kaplan Meier method. There was a significant difference in survival between included cases and those excluded due to missing clinical or imaging data for the anti-PD1 therapy group (OS HR 0.45 95% CI [0.32–0.65] p<0.001, TTNT HR 0.46 95% CI [0.32–0.65] p<0.001) and the ipilimumab-nivolumab group (OS HR 0.50 95% CI [0.26–0.93] p=0.030, TTNT HR 0.38 95% CI [0.22–0.66] p=0.001) as seen in **Supplementary Figure 2**.

Baseline participant characteristics at the time of first-line ICI therapy initiation as stratified by objective imaging response are demonstrated in **Table 1**. A total of 127 patients (64.1%) experienced a response, while 71 patients (35.9%) did not. Baseline variables including median age (59 years IQR [52–71] vs 63 years IQR [52, 75]), biological sex (male, 44/71 [62%] vs 86/127 [67.7%]), and stage (stage IV, 63/71 [88.7%] vs 111/127 [87.4%]) were similar between non-responders and responders. In comparison to responders, non-responders were more likely to have a mucosal primary site (11/71 [15.5%] vs 4/1127 [3.1%]; p=0.006), have a worse ECOG performance status (ECOG <1, 28/71 [39.4%] vs 69/127 [54.3%]), and have a lactate dehydrogenase level above the upper limit of normal (26/71 [36.6%] vs 25/127 [19.7%]; p=0.009). There was no difference in the types of ICI therapy received between non-responders and responders in our cohort (p=0.85).

**Table 1:** Baseline participant characteristics by objective imaging response.

	Non-Responder (N=71)	Responder (N=127)	P-Value
**Age, Median (IQR)**	59 (52,71)	63 (52,75)	0.224
**Sex, Male (%)**	44 (62)	86 (67.7)	0.414
Primary Site (%)			
Cutaneous	47 (66.2)	101 (79.5)	0.006
Mucosal	11 (15.5)	4 (3.1)	
Unknown	13 (18.3)	22 (17.3)	
ECOG Performance Status (%)			
ECOG < 1	28 (39.4)	69 (54.3)	0.044
ECOG ≥ 1	43 (60.6)	58 (45.7)	
Stage at ICI Therapy Initiation (%)			
III*	5 (7)	8 (6.3)	0.59
IIIB	0 (0)	4 (3.1)	
IIIC	3 (4.2)	4 (3.1)	
IV	63 (88.7)	111 (87.4)	
Liver Metastases (%)			
Absent	49 (69)	100 (78.7)	0.13
Present	22 (31)	27 (21.3)	
Brain Metastases (%)			
Absent	59 (83.1)	113 (89)	0.24
Present	12 (16.9)	14 (11)	
Bone Metastases (%)			
Absent	54 (76.1)	105 (82.7)	0.26
Present	17 (23.9)	22 (17.3)	
BRAF Mutation (%)			
Absent	54 (76.1)	109 (85.8)	0.08
Present	17 (23.9)	18 (14.2)	
ICI Therapy (%)			
Nivolumab	13 (18.3)	20 (15.7)	0.85
Pembrolizumab	33 (46.5)	58 (45.7)	
Ipilimumab-Nivolumab	25 (35.2)	49 (38.6)	
LDH Level (%)			
LDH ≤ ULN	45 (63.4)	102 (80.3)	0.009
LDH > ULN,	26 (36.6)	25 (19.7)	
Albumin Level (%)			
Albumin ≥ LLN	64 (86.5)	104 (83.9)	0.03
Albumin < LLN	10 (13.5)	20 (16.1)	
WBC Level (%)			
WBC ≤ 11	66 (89.2)	114 (91.9)	0.78
WBC > 11	8 (10.8)	10 (8.1)	

^*^ Unknown if Stage IIIA, IIIB, or IIIC.

ECOG, Eastern Cooperative Oncology Group; ICI, Immune checkpoint Inhibitor; WBC, white blood cells; ULN, upper limit of normal; LLN, lower limit of normal.

### Baseline patient characteristics and objective response

**Figure 1:**
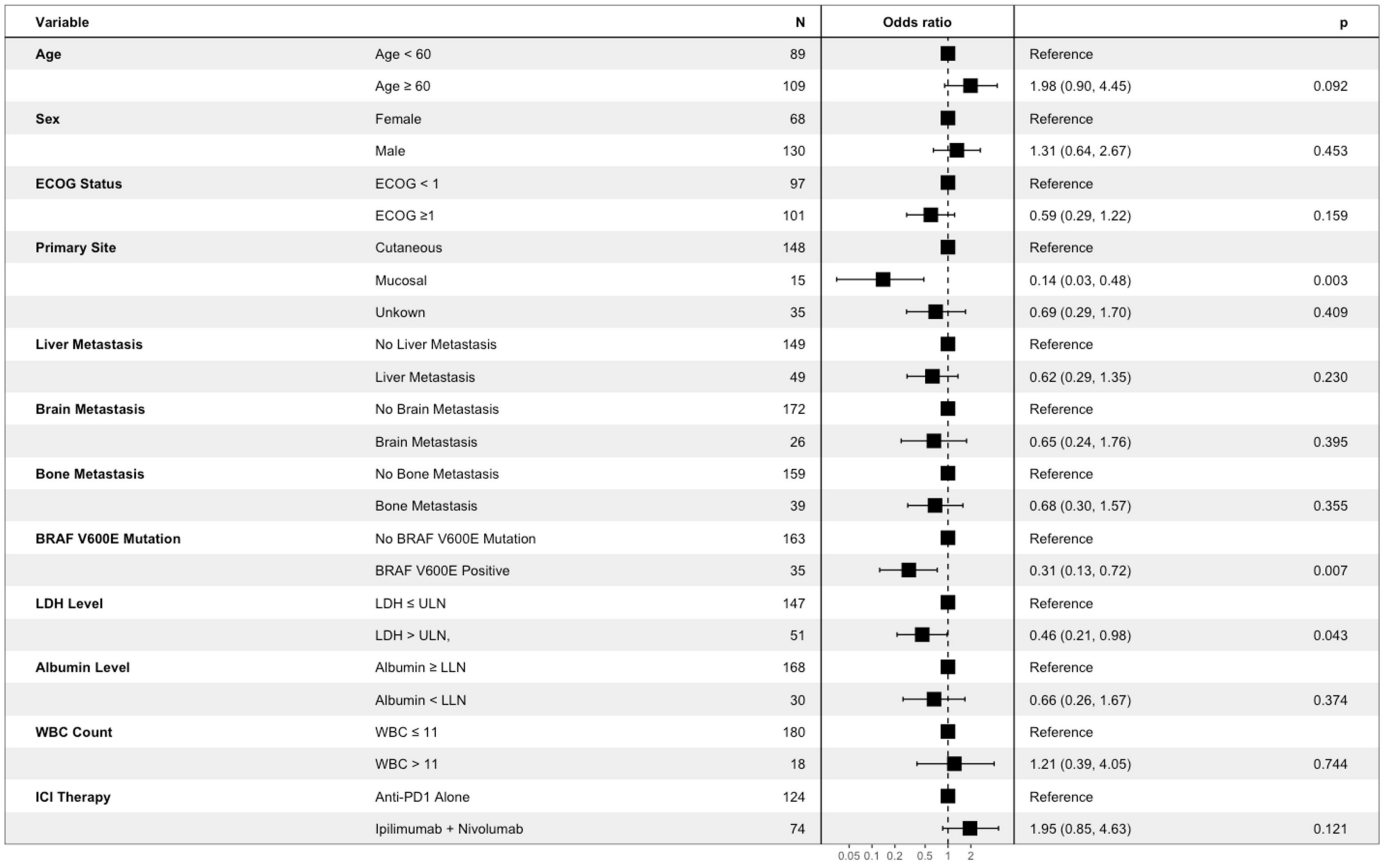
Association between baseline participant characteristics of interest and objective imaging response of patients with advanced melanoma receiving first line immune checkpoint inhibitor therapy. Results are from multivariable logistic regression analysis. Box represents point estimate of Odds Ratio, and whiskers represent Wald 95% confidence interval.

To identify routinely collected baseline characteristics associated with likelihood of objective imaging response, logistic regression was conducted. Baseline characteristics of interest were identified *a priori* based upon established or probable association with survival endpoints and/or imaging response. These included age, biological sex, primary site, ECOG status, BRAF V600E mutation status, LDH level, albumin level, white blood cell count, and presence of liver, bone, and brain metastases (30). Also included in this analysis was ICI therapy received (anti-PD1 monotherapy vs ipilimumab-nivolumab), to assess if choice of therapy has an impact on likelihood of imaging response. Of the variables included, LDH level above the upper limit of normal (OR 0.46; 95% CI 0.21–0.98, p=0.043), mucosal primary site (OR 0.14; 95% CI 0.03–0.48, p=0.003), and BRAF V600E mutation status (OR 0.31; 95% CI 0.13–0.72, p=0.007), were associated with significantly decreased odds of objective imaging response (**Figure 1**). Treatment with anti-PD1 monotherapy (nivolumab or pembrolizumab) vs treatment with ipilimumab-nivolumab was not associated with increased likelihood of objective imaging response (OR 1.95 95% CI [0.85–4.63], p=0.121) in this analysis (**Figure 1**).

### Relationship between objective imaging response and overall survival

**Figure 2:**
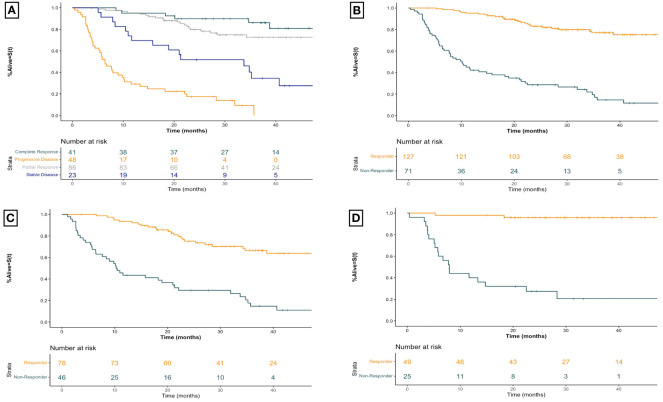
Kaplan-Meier overall survival curves for advanced melanoma patients receiving first-line immune checkpoint inhibitor therapy for **(A)** all patients receiving first-line ICI therapy by best overall imaging response **(B)** all patients receiving first-line ICI therapy by imaging response **(C)** patients receiving first-line anti-PD1 therapy by imaging response, and **(D)** patients receiving first-line ipilimumab-nivolumab by imaging response. Plus signs represent censoring time. X-axis for all curves is time since initiation of treatment in months. Responders include patients with a best overall objective imaging response of complete or partial response. Non-responders include patients with a best overall objective imaging response of stable or progressive disease. Anti-PD1 therapy includes those patients treated with anti-PD1 monotherapy with nivolumab or pembrolizumab.

The overall association between BOR and OS is demonstrated in **Figure 2A**. The median OS was not reached (NR) (95% CI 49.0-NR) months for CR, NR (95% CI 52.9-NR) months for PR, 33.7 (95% CI 15.8-NR) months for SD, and 6.4 (95% CI 5.2–10.1) months for PD (log rank p<0.001). Categorizing patients into responders vs non-responders (**Figure 2B**) demonstrated a median OS of NR (95% CI 52.9-NR) months vs 10.1 (95% CI 7.7–18.2) months respectively (HR 7.47 95% CI [4.74–11.80], p<0.001).

The strong association between imaging response and survival is seen for those receiving first-line anti-PD1 therapy as well (**Figure 2C**). Responders had a median OS of NR (95% CI 49.0-NR) months compared to 10.4 (95% CI 7.7–21.3) months for non-responders (HR 4.99 95% CI [2.98–8.33], p<0.001). For patients receiving combination ipilimumab-nivolumab, seen in **Figure 2D**, responders had a median OS of NR (95% CI 52.9-NR) months compared to non-responders who experienced a median OS of 7.9 (95% CI 5.8– 28.3) months (HR 22.29 95% CI [6.52–76.19], p<0.001). Similar robust associations are seen when patients are stratified by BOR across both therapy categories (**Table 2**).

**Table 2:** Overall survival and time to next treatment outcomes by best overall imaging response among patients with advanced melanoma treated with first line immune checkpoint inhibitor therapies.

Therapy Received	Best Overall Response No. (%)	Overall Survival		Time to Next Treatment	
		Median OS mo. (95% CI)	2-year OS % (95% CI)	Median TTNT mo. (95% CI)	2-year TTNT % (95% CI)
Single Agent anti-PD1 Therapy (n=124)	Complete Response 26 (21.0)	NE (49.0 – NE)	87.6 (75.4 – 100.0)	NE (49.0-NE)	87.6 (75.4 – 100.0)
	Partial Response 52 (41.9)	NE (28.5 – NE)	69.0 (57.1– 83.5)	50.5 (23.2 – NE)	61.5 (49.2 – 76.9)
	Stable Disease 18 (14.5)	33.7 (18.9 – NE)	55.0 (36.0 – 83.9)	20.0 (15.8 – NE)	38.1 (20.9 – 69.3)
	Progressive Disease 28 (22.6)	6.1 (3.5 – 10.3)	12.9 (4.7 – 35.2)	5.6 (3.5 – 9.0)	3.6 (0.5 – 24.5)
Combination Ipilimumab-Nivolumab Therapy (n= 74)	Complete Response 15 (20.3)	NE (NE – NE)	93.3 (81.5 – 100.0)	NE (NE – NE)	93.3 (81.5 – 100.0)
	Partial Response 34 (45.9)	NE (52.9 – NE)	64.7 (29.0 – 100.0)	NE (NE – NE)	85.3 (74.2 – 98.1)
	Stable Disease 5 (6.8)	11.7 (7.9 – NE)	40.0 (13.7 – 100.0)	10.7 ( 5.1 – NE)	40.0 (13.7 – 100.0)
	Progressive Disease 20 (27.0)	7.3 (5.2 – 28.3)	24.0 (10.8 - 53.4)	3.7 (3.1 – 5.9)	5.0 (0.7 – 33.8)

### Relationship between objective imaging response and time to next treatment

**Figure 3:**
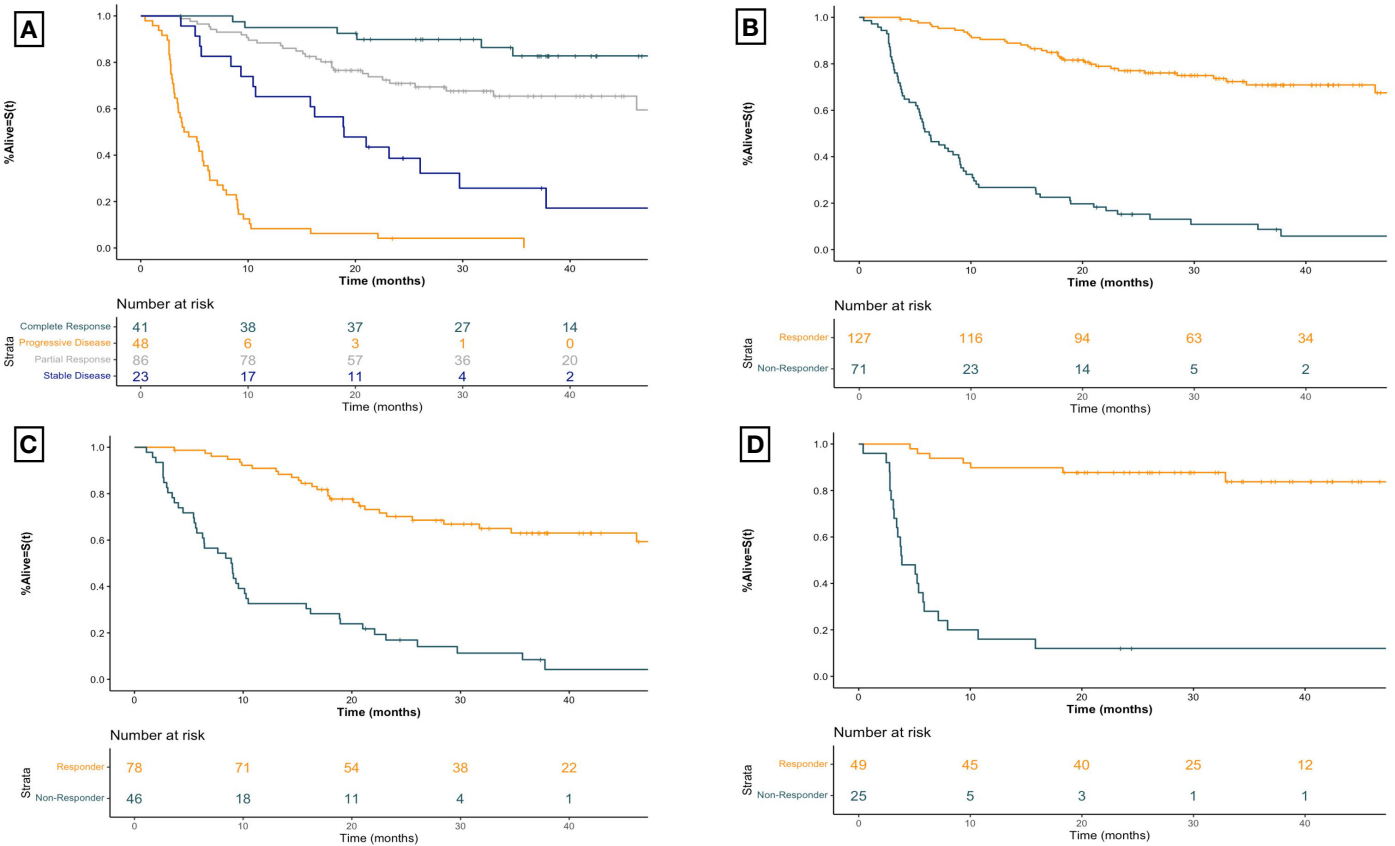
Kaplan-Meier time to next treatment curves for advanced melanoma patients receiving first-line immune checkpoint inhibitor therapy for **(A)** all patients receiving first-line ICI therapy by best overall imaging response **(B)** all patients receiving first-line ICI therapy by imaging response **(C)** patients receiving first-line anti-PD1 therapy by imaging response, and **(D)** patients receiving first-line ipilimumab-nivolumab by imaging response. Plus signs represent censoring time. X-axis for all curves is time since initiation of treatment in months. Responders include patients with a best overall objective imaging response of complete or partial response. Non-responders include patients with a best overall objective imaging response of stable or progressive disease. Anti-PD1 therapy includes those patients treated with anti-PD1 monotherapy with nivolumab or pembrolizumab.

The relationship between BOR and TNTT is shown in **Figure 3A**. The median TTNT for CR was NR (95% CI NR-NR) months, compared to NR (95% CI 46.2-NR) months for PR, 18.9 (95% CI 10.7-NR) months for SD, and 4.3 (95% CI 3.5–6.3) months for PD. Comparison between responders and non-responders, seen in **Figure 3B**, demonstrated a similar association with TTNT, with responders having a longer median TTNT of NR (95% CI 50.5-NR) months compared to 6.3 (95% CI 5.4–9.0) months for non-responders (HR 8.11 95% CI [5.32–12.36], p<0.001).

Within the anti-PD1 monotherapy group (**Figure 3C**) responders had a median TTNT of NR (95% CI 46.2-NR) months compared to non-responders who experienced a median TTNT of 9.0 (95% CI 6.3–10.5) months (HR 5.89 95% CI [3.60–9.64], p<0.001). Similarly, for patients receiving combined ipilimumab-nivolumab therapy (**Figure 3D**) the median TTNT for responders was NR (95% CI NR-NR) months compared to non-responders who had a median TTNT of 3.9 (95% CI 3.4–7.1) months (HR 16.05 95% CI [6.66–38.69], p<0.001). This strong association between imaging response and TTNT regardless of therapy regimen was demonstrated by BOR as well (**Table 2**).

## Discussion

To our knowledge, this observational cohort study represents the largest observational analysis of patients with locally advanced or metastatic melanoma receiving first-line ICI therapy to characterize the association of objective imaging response with baseline patient characteristics as well as with key survival outcomes. Though numerous studies have examined the relationship between baseline patient characteristics and survival outcomes, we are among the first to examine how these may relate to the likelihood of objective imaging response, a crucial gap in the literature. A better understanding of which patients may have an objective imaging response, and what that imaging response may mean for their long-term survival outcomes is crucial for proper counseling of patients and treatment decision making.

Our results demonstrate that investigator assessed imaging response has a strong association with overall survival and time to next treatment outcomes in advanced melanoma patients treated with first-line ICI therapy. These associations remain robust when examining patients by therapy type and when grouping patients based on RECIST v1.1 defined BOR. In addition to demonstrating this relationship in a routine practice setting, we also provide survival estimates by therapy type and best overall imaging response, which may be useful to council patients with advanced melanoma treated with these therapies in a real-world setting. The remarkable plateau in overall survival seen in patients that respond to ipilimumab-nivolumab (**Figure 2D**) compared to those that respond to PD-1 monotherapy (**Figure 2C**), are particularly relevant to inform patients about when counselling between these agents.

We further characterized the likelihood of objective imaging response between contemporary first-line ICI therapies for advanced melanoma. In this analysis, those receiving combined ipilimumab-nivolumab were not more likely to have an objective imaging response compared to those receiving anti-PD1 monotherapy (OR 1.95 95% CI 0.85–4.63, p=0.121) (**Figure 1**). We found that a similar proportion of patients having objective imaging response to single agent anti-PD1 therapy (78/124, 62.9%) compared to combination ipilimumab-nivolumab (49/74, 66.2%) (**Table 2**). These results are similar to a recently published single-center cohort study which demonstrated similar rates of CR between anti-PD1 monotherapy and combination ipilimumab-nivolumab (31). These objective imaging response rates are similar to those reported in clinical trials evaluating combined ipilimumab-nivolumab (4, 18) and marginally increased compared to those reported in trials evaluating single agent nivolumab or pembrolizumab (14–17) in advanced melanoma patients. However, these results should be interpreted with caution as the potential for treatment selection bias present in observational cohort studies makes formal statistical comparisons challenging. In particular, individuals receiving ipilimumab-nivolumab were more likely to be BRAF V600E mutation positive, have brain metastasis, be younger, and have an ECOG performance status <1 (**Supplementary Table 1**). These imbalances are emblematic of the bias that occurs in the selection of therapies for patients in a routine practice setting and demonstrates the possibility of differences in other important co-variates between groups, not accounted for in our work, which may have affected the observed relationship between therapy and imaging response. Additionally, low sample size may have contributed to this finding. As an example, utilizing the objective response rate of 45% for nivolumab monotherapy and 58% for ipilimumab-nivolumab therapies reported in a previous landmark clinical trial (20), a sample size of 462 patients would be required to detect differences at a type II error rate of <0.20. The sample size of this study (n=198) was comparatively modest, possibly accounting for why our results deviate from those reported previously.

We further demonstrate that BRAF V600E mutation status, an elevated LDH above the laboratory reported upper limit of normal, and mucosal histology were all associated with decreased likelihood of objective imaging response to first-line ICI therapies. The prognostic importance of the BRAF V600E mutation in advanced melanoma remains unclear, with multiple studies having variably linked its presence to survival outcomes (32–35), with a recent meta-analysis demonstrating significant heterogeneity between studies (36). There have been very few studies investigating BRAF V600E mutation status and odds of objective imaging response to ICI (37), and to our knowledge we are among the first to report this association in a real-world cohort of patients with advanced melanoma receiving first-line ICI therapy.

Elevated LDH levels have been associated with poor survival and decreased objective response rate in advanced melanoma patients across multiple clinical trial settings (4, 38–40). This baseline biochemical test has also been included in recent prognostic scoring models for patients with metastatic melanoma treated with ICIs (30). There are multiple mechanisms postulated for this association, including increased LDH activity being a marker of glycolytic activity or elevated levels of hypoxia-mediated tumour necrosis, both of which are associated with increased tumour burden (41). Therefore, these results are in line with other evidence associating elevated LDH levels with decreased likelihood of objective imaging response and bolsters this body of evidence by demonstrating this association for patients.

Mucosal melanomas are a relatively rare subtype, representing close to 1% of all melanoma diagnoses (42). These cancers are associated with a higher mortality rate and poorer prognosis than their cutaneous counterparts, largely owing to presentation at later stages (43). There are currently no specific treatment guidelines for patients with advanced mucosal melanoma, and therefore first-line ICI therapies remain standards of care. Due to their relative scarcity, the majority of data regarding objective response rate to ICI therapy comes from pooled analyses of clinical trials. Regardless, results of these analysis have shown that mucosal melanoma patients experience poorer objective response rates to first-line ICI therapies than those with cutaneous melanomas (43, 44), concordant with our real-world findings.

This study has numerous strengths that add value to the literature surrounding objective imaging response in the setting of advanced melanoma. Our results are based on the study of a large patient cohort treated in a real-world setting, which bolsters the clinical relevance and translatability of these results to the routine clinical practice setting. Additionally, we examined OS and TTNT, survival outcomes of importance to patients, over a relatively long median follow up period. This combination allows insights that oncologists can utilize to better counsel patients and set appropriate expectations for treatment outcomes. Additionally, given that the pivotal ipilimumab-nivolumab trial did not formally statistically compare the doublet regimen with nivolumab monotherapy (11), this work also adds context to the real-world differences between outcomes when these agents are used.

This study also has limitations that should be considered. With regards to assessment of RECIST v1.1 imaging response, it is important to note that these were done in a routine-practice setting without blinded independent centralized review, and at inconsistent intervals between patients. Therefore, these may not adhere strictly to the rigorous guidelines recommended for reporting objective response in the clinical trial setting. Additionally, the uneven timing of baseline and repeat re-staging imaging may introduce an element of informative censoring to the results presented.

Moreover, there were significant differences in these outcomes between patients included and excluded in this study (**Supplementary Figure 2**), suggesting potential imbalances between cohorts. When the shape of the survival curves in **Supplementary Figure 2** are reviewed, a substantial number of patients in the excluded group experienced an event within 3 months, likely precluding them from having an imaging response assessment scan, thereby resulting in their exclusion from the primary analysis. The exclusion of these patients may have influenced the survival estimates reported in this study. In addition, differences in baseline characteristics, such as a greater proportion of patients with an elevated LDH, may also have contributed to poorer outcomes for those excluded from the initial analysis (**Supplementary Table 2**).

Additionally, though multivariable regression incorporating baseline clinical, pathological, and laboratory variables was utilized in the comparison between single agent anti-PD1 therapies and combination ipilimumab-nivolumab, numerous cofounders not accounted for this this analysis may have affected the results obtained. The differences in baseline characteristics between single agent ati-PD1 therapies and combination ipilimumab-nivolumab likely reflects the non-random selection of patients which occurs in routine practice settings. Moreover, the patients included in this study were treated in a single province, at both academic and community centers, which may limit the generalizability of our findings to other treatment settings, including other jurisdictions or practice types.

We present the among the first real-world observational data to examine objective imaging response and survival outcomes in advanced melanoma patients treated with first-line ICI therapy. Our findings suggest that objective imaging response was associated with improved OS and TTNT for advanced melanoma patients regardless of treatment regimen. We also demonstrate that though the likelihood of response does not vary based on type of ICI therapy received, that the presence of BRAF V600E mutation status, elevated LDH levels, and mucosal subsite are all associated with decreased odds of an objective imaging response. These findings may help inform treatment decisions in the context of high-volume metastatic disease in which obtaining objective imaging response is important and may aid oncologists in counseling patients treated first-line ICI therapy regarding treatment outcomes.

## Data availability statement

The raw data supporting the conclusions of this article will be made available by the authors, without undue reservation.

## Ethics statement

This study was approved by the Health Research Ethics Board of Alberta-Cancer Committee (HREBA.CC-19–0380). The studies were conducted in accordance with the local legislation and institutional requirements. Written informed consent for participation was not required from the participants or the participants’ legal guardians/next of kin in accordance with the national legislation and institutional requirements.

## Author contributions

MG: Conceptualization, Data curation, Formal analysis, Investigation, Validation, Visualization, Writing – original draft, Writing – review & editing. IS: Conceptualization, Data curation, Investigation, Writing – review & editing. DM: Conceptualization, Data curation, Investigation, Writing – review & editing. DH: Conceptualization, Data curation, Investigation, Methodology, Project administration, Resources, Supervision, Writing – review & editing. JM: Conceptualization, Investigation, Project administration, Resources, Supervision, Writing – review & editing. TC: Conceptualization, Data curation, Methodology, Project administration, Resources, Supervision, Writing – review & editing. VN: Conceptualization, Data curation, Investigation, Methodology, Project administration, Resources, Supervision, Writing – original draft, Writing – review & editing.
