# Supplementary material for: Imaging response to immune checkpoint inhibitors in patients with advanced melanoma: a retrospective observational cohort study
Source: Front Oncol. 2024 May 31;14:1385425. doi: 10.3389/fonc.2024.1385425 (PMC11176500; doi:10.3389/fonc.2024.1385425)
Supplement: Supplementary file 1 [file Presentation_1.pptx]

## Slide 1
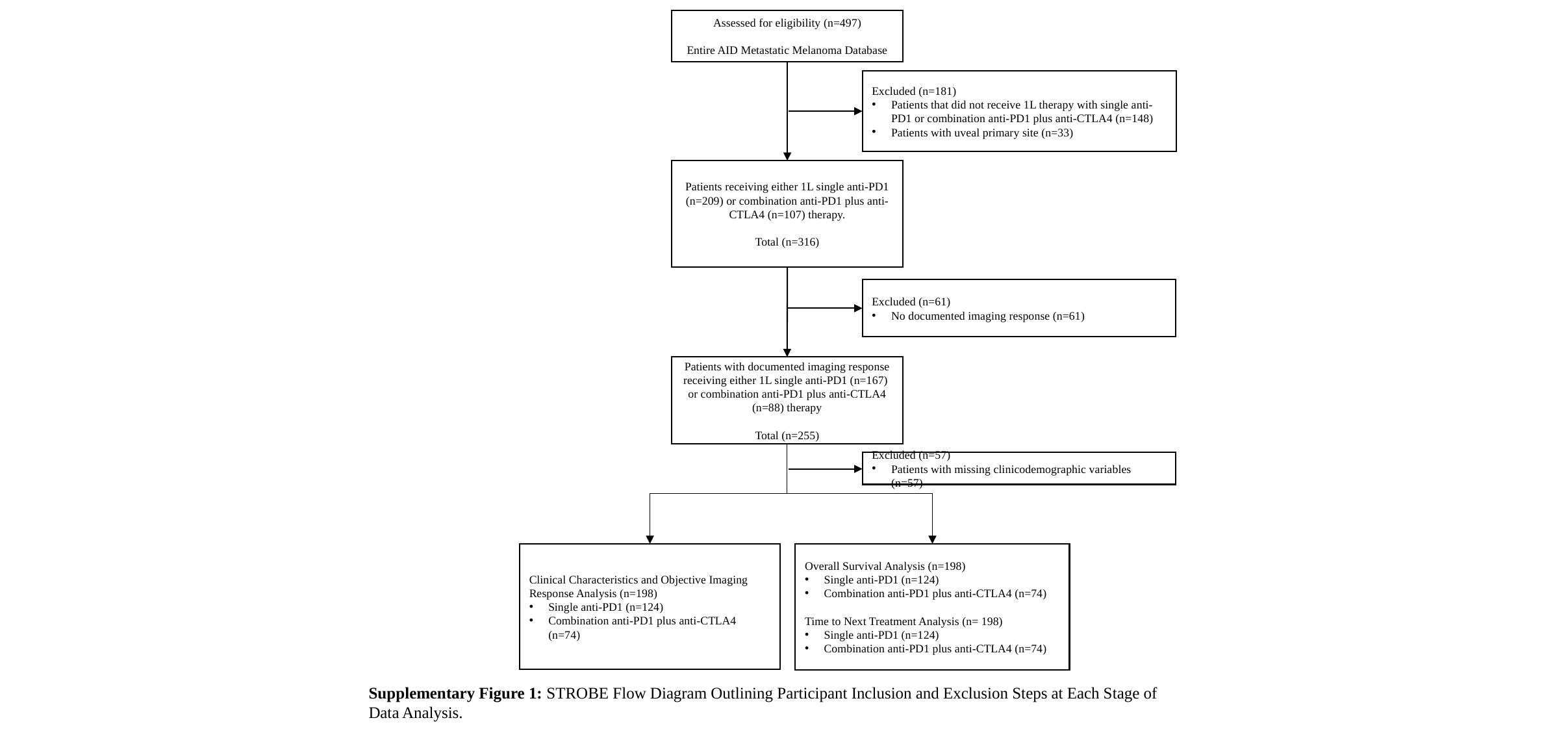

Assessed for eligibility (n=497)
Entire AID Metastatic Melanoma Database
Excluded (n=181)
Patients that did not receive 1L therapy with single anti-PD1 or combination anti-PD1 plus anti-CTLA4 (n=148)
Patients with uveal primary site (n=33)
Patients receiving either 1L single anti-PD1 (n=209) or combination anti-PD1 plus anti-CTLA4 (n=107) therapy.
Total (n=316)
Excluded (n=61)
No documented imaging response (n=61)
Patients with documented imaging response receiving either 1L single anti-PD1 (n=167)
or combination anti-PD1 plus anti-CTLA4 (n=88) therapy
Total (n=255)
Excluded (n=57)
Patients with missing clinicodemographic variables (n=57)
Clinical Characteristics and Objective Imaging Response Analysis (n=198)
Single anti-PD1 (n=124)
Combination anti-PD1 plus anti-CTLA4 (n=74)
Overall Survival Analysis (n=198)
Single anti-PD1 (n=124)
Combination anti-PD1 plus anti-CTLA4 (n=74)
Time to Next Treatment Analysis (n= 198)
Single anti-PD1 (n=124)
Combination anti-PD1 plus anti-CTLA4 (n=74)
Supplementary Figure 1: STROBE Flow Diagram Outlining Participant Inclusion and Exclusion Steps at Each Stage of Data Analysis.
