# Supplementary material for: Imaging response to immune checkpoint inhibitors in patients with advanced melanoma: a retrospective observational cohort study
Source: Front Oncol. 2024 May 31;14:1385425. doi: 10.3389/fonc.2024.1385425 (PMC11176500; doi:10.3389/fonc.2024.1385425)
Supplement: Supplementary file 2 [file Presentation_2.pptx]

## Slide 1
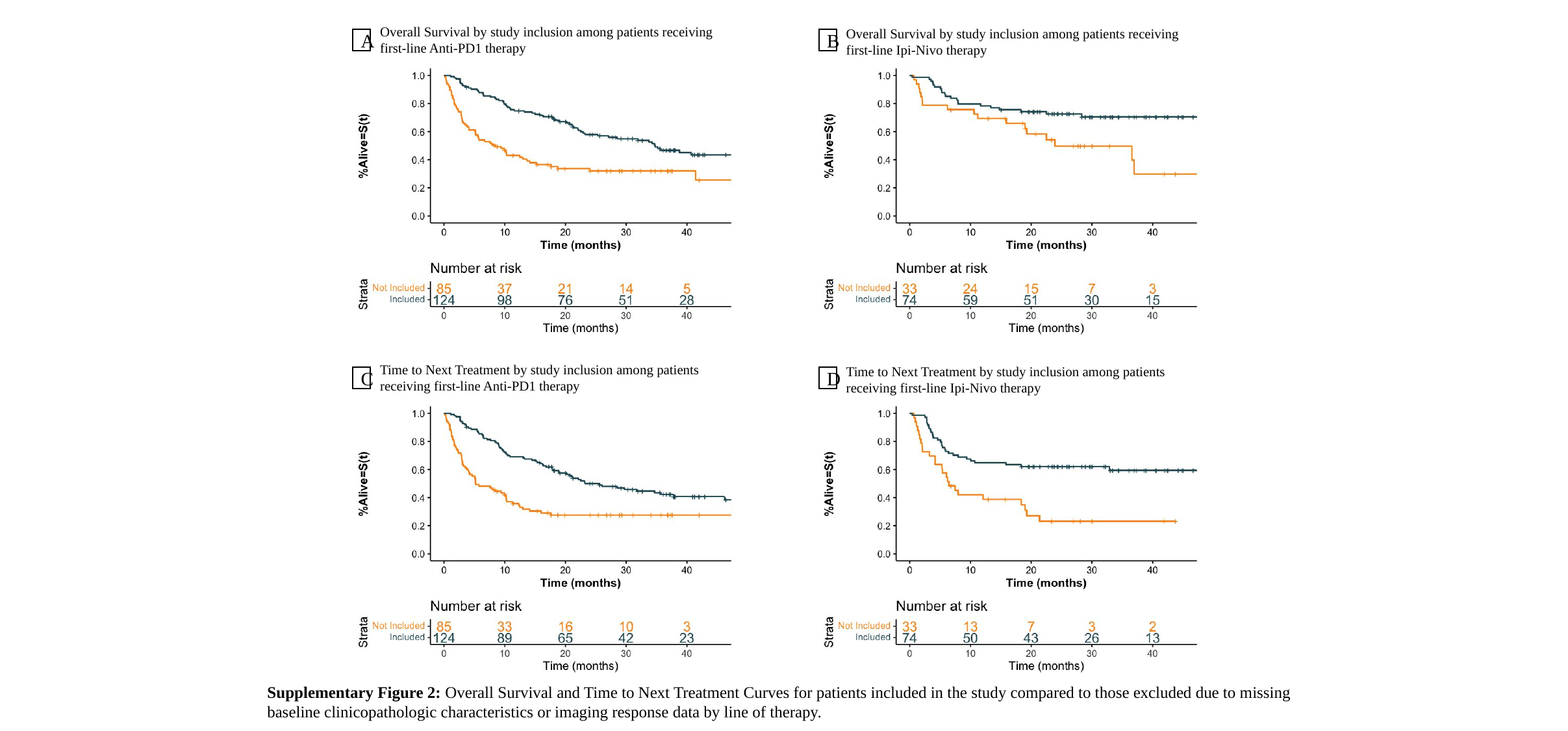

Overall Survival by study inclusion among patients receiving first-line Anti-PD1 therapy
Overall Survival by study inclusion among patients receiving first-line Ipi-Nivo therapy
A
B
Time to Next Treatment by study inclusion among patients receiving first-line Anti-PD1 therapy
Time to Next Treatment by study inclusion among patients receiving first-line Ipi-Nivo therapy
C
D
Supplementary Figure 2: Overall Survival and Time to Next Treatment Curves for patients included in the study compared to those excluded due to missing baseline clinicopathologic characteristics or imaging response data by line of therapy.
