# Supplementary material for: Imaging response to immune checkpoint inhibitors in patients with advanced melanoma: a retrospective observational cohort study
Source: Front Oncol. 2024 May 31;14:1385425. doi: 10.3389/fonc.2024.1385425 (PMC11176500; doi:10.3389/fonc.2024.1385425)
Supplement: Supplementary file 3 [file Table_1.docx]

**Supplementary Table 1:** Baseline Participant Characteristics by type of first-line immune checkpoint inhibitor therapy received

|  | **Ipilimumab-Nivolumab (N=74)** | **Single agent anti-PD1**  **(N=124)** | **P value** |
| --- | --- | --- | --- |
| **Age, Median (IQR)** | 52.5 (42.2,59) | 70 (59,78) | < 0.001 |
| **Sex, Male (%)** | 51 (68.9) | 79 (63.7) | 0.455 |
| **ECOG Performance Status (%)** | | | |
| ECOG < 1 | 44 (59.5) | 53 (42.7) | 0.023 |
| ECOG ≥ 1 | 30 (40.5) | 71 (57.3) |  |
| **Stage at ICI Therapy Initiation (%)** | | | |
| III* | 6 (8.1) | 7 (5.6) | 0.513 |
| IIIB | 2 (2.7) | 2 (1.6) |  |
| IIIC | 4 (5.4) | 3 (2.4) |  |
| IV | 62 (83.8) | 112 (90.3) |  |
| **Primary Site (%)** | | | |
| Cutaneous | 57 (77) | 91 (73.4) | 0.12 |
| Mucosal | 2 (2.7) | 13 (10.5) |  |
| Other | 15 (20.3) | 20 (16.1) |  |
| **Brain Metastases (%)** | | | |
| No Brain Metastasis | 59 (79.7) | 113 (91.1) | 0.022 |
| Brain Metastasis | 15 (20.3) | 11 (8.9) |  |
| **Bone Metastases (%)** | | | |
| No Bone Metastasis | 61 (82.4) | 98 (79) | 0.561 |
| Bone Metastasis | 13 (17.6) | 26 (21) |  |
| **Liver Metastases (%)** | | | |
| No Liver Metastasis | 58 (78.4) | 91 (73.4) | 0.431 |
| Liver Metastasis | 16 (21.6) | 33 (26.6) |  |
| **BRAF Mutation (%)** | | | |
| BRAF V600E Positive | 23 (31.1) | 12 (9.7) | < 0.001 |
| No BRAF V600E Mutation | 51 (68.9) | 112 (90.3) |  |
| **LDH Level (%)** | | | |
| LDH ≤ ULN | 50 (67.6) | 97 (78.2) | 0.097 |
| LDH > ULN, | 24 (32.4) | 27 (21.8) |  |
| **Albumin Level (%)** | | | |
| Albumin ≥ LLN | 64 (86.5) | 104 (83.9) | 0.619 |
| Albumin < LLN | 10 (13.5) | 20 (16.1) |  |
| **WBC Level (%)** | | | |
| WBC ≤ 11 | 66 (89.2) | 114 (91.9) | 0.515 |
| WBC > 11 | 8 (10.8) | 10 (8.1) |  |

^*^ Unknown if Stage IIIA, IIIB, or IIIC

Abbreviations: ECOG, Eastern Cooperative Oncology Group; ICI, Immune checkpoint Inhibitor; WBC, white blood cells; LDH, lactate dehydrogenase
