# Supplementary material for: Imaging response to immune checkpoint inhibitors in patients with advanced melanoma: a retrospective observational cohort study
Source: Front Oncol. 2024 May 31;14:1385425. doi: 10.3389/fonc.2024.1385425 (PMC11176500; doi:10.3389/fonc.2024.1385425)
Supplement: Supplementary file 4 [file Table_2.docx]

**Supplementary Table 2:** Baseline Participant Characteristics by Study Inclusion

|  | **Included (N=198)** | **Excluded (N=118)^#^** | **P value** |
| --- | --- | --- | --- |
| **Age, Median (IQR)** | 62.0 (52,73) | 64.5 (55.5,73) | 0.258 |
| **Sex, Male (%)** | 130 (65.7) | 85 (72) | 0.240 |
| **ECOG Performance Status (%)** | | | |
| ECOG < 1 | 97 (49) | 46 (39.3) | 0.096 |
| ECOG ≥ 1 | 101 (51) | 71 (60.7) |  |
| **Stage at ICI Therapy Initiation (%)** | | | |
| III* | 14 (11.9) | 13 (6.6) | 0.087 |
| IIIA | 1 (0.8) | 0 (0) |  |
| IIIB | 4 (3.4) | 4 (2) |  |
| IIIC | 8 (6.8) | 7 (3.5) |  |
| IV | 91 (77.1) | 174 (87.9) |  |
| **Primary Site (%)** | | | |
| Cutaneous | 148 (74.7) | 90 (76.3) | 0.947 |
| Mucosal | 15 (7.6) | 8 (6.8) |  |
| Other | 35 (17.7) | 20 (16.9) |  |
| **Brain Metastases (%)** | | | |
| Brain Metastasis | 26 (13.1) | 17 (14.4) | 0.749 |
| No Brain Metastasis | 172 (86.9) | 101 (85.6) |  |
| **Bone Metastases (%)** | | | |
| Bone Metastasis | 39 (19.7) | 17 (14.4) | 0.234 |
| No Bone Metastasis | 159 (80.3) | 101 (85.6) |  |
| **Liver Metastases (%)** | | | |
| Liver Metastasis | 49 (24.7) | 36 (30.5) | 0.264 |
| No Liver Metastasis | 149 (75.3) | 82 (69.5) |  |
| **BRAF Mutation (%)** | | | |
| BRAF V600E Positive | 35 (17.7) | 18 (15.3) | 0.577 |
| No BRAF V600E Mutation | 163 (82.3) | 100 (84.7) |  |
| **LDH Level (%)** | | | |
| LDH ≤ ULN | 147 (74.2) | 30 (51.7) | 0.001 |
| LDH > ULN, | 51 (25.8) | 28 (48.3) |  |
| **Albumin Level (%)** | | | |
| Albumin ≥ LLN | 168 (84.8) | 39 (65) | < 0.001 |
| Albumin < LLN | 30 (15.2) | 21 (35) |  |
| **WBC Level (%)** | | | |
| WBC ≤ 11 | 180 (90.9) | 84 (83.2) | 0.049 |
| WBC > 11 | 18 (9.1) | 17 (16.8) |  |

Abbreviations: ECOG, Eastern Cooperative Oncology Group; ICI, Immune checkpoint Inhibitor; WBC, white blood cells; LDH, lactate dehydrogenase

^*^Unknown if Stage IIIA, IIIB, or IIIC

^#^ Note: A subset of patients were excluded from this study due to missing clinicodemographic characteristics. Therefore, the total number of patients in any particular demographic category for the excluded group may not sum to the total of 118 as this information is unknown.
